# Composition and structure of the marine benthic community in Terra Nova Bay, Antarctica: Responses of the benthic assemblage to disturbances

**DOI:** 10.1371/journal.pone.0225551

**Published:** 2019-12-02

**Authors:** Yun Hee Kang, Sanghee Kim, Sun Kyeong Choi, Kyeonglim Moon, Han-Gu Choi, Young Wook Ko, Ian Hawes, Sa-Heung Kim, Ji Hee Kim, Sang Rul Park

**Affiliations:** 1 Department of Earth and Marine Sciences, Jeju National University, Jeju, Republic of Korea; 2 Department of Polar Life Sciences, Korea Polar Research Institute, Incheon, Republic of Korea; 3 Estuarine & Coastal Ecology Laboratory, Department of Marine Life Sciences, Jeju National University, Jeju, Republic of Korea; 4 Coastal Marine Field Station, University of Waikato, Sulphur Point, Tauranga, New Zealand; 5 Marine Biodiversity Research Institute, INTHESEA KOREA Inc., Jeju, Republic of Korea; CIIMAR Interdisciplinary Centre of Marine and Environmental Research of the University of Porto, PORTUGAL

## Abstract

The community structure and assemblages of marine benthic organisms were investigated in coastal areas near the Jang Bogo Antarctic Research Station in Terra Nova Bay during the 2012–2018 summer seasons. We also examined the recovery pattern of marine benthic organisms following disturbance due to the construction of the Jang Bogo Station. A total of 26 taxa were identified in the study area during the experimental period. Species number and diversity indices (richness, evenness, and diversity) were relatively low compared to data previously reported from Terra Nova Bay. *Sphaerotylus antarcticus*, *Clavularia frankliniana*, *Hydractinia* sp., *Iridaea cordata*, *Fragilariopsis* spp., *Alcyonium antarcticum*, and *Metalaeospira pixelli* were the dominant species in this area. Of these, the diatom *Fragilariopsis* spp. were the most abundant species, indicating their key role in maintaining the marine benthic community and controlling biogeochemical cycling. During the construction of the Jang Bogo Station, sediment coverage increased and diatoms declined due to the release of sediment into the coastal area. In February 2014, one month after the disturbance due to cyclone, the diatom coverage increased dramatically and thereby species number, richness index, and diversity index steadily rose from 2015 to 2018. However, non-metric multidimensional scaling ordination analysis of species similarities among sampling times showed that community structure had not completely recovered by 2018. Thus, long-term monitoring is required to elucidate the post-disturbance settlement mechanisms of marine benthic organisms at the study area in Terra Nova Bay.

## Introduction

Antarctica, Earth's southernmost continent, is approximately 14.2 million km^2^ and is almost completely covered by an ice sheet. The Antarctic marine environment is one of the most thermally stable on earth, with ocean temperatures exhibiting an annual range of only 3–4°C [1,2]. However, parts of Antarctica have emerged as among the most rapidly warming regions on Earth over the last half century [3]. Many studies in Antarctica have focused on biological responses to such climate change, including studies of the response of the nearshore benthos to increasing temperatures [3–5]. Although the abundance and distribution of specific species can respond rapidly to disturbance, changes in the marine benthic ecosystem due to such factors can be expected to occur gradually over a long period of time. Many ecological processes, such as growth and recruitment rates, occur more slowly in Antarctica than in temperate and tropical regions [6]. Thus, several marine Long-Term Ecological Research (LTER) studies have been conducted for various purposes [7–10]. A few long-term, comprehensive studies have been conducted on the dynamics and structure of Antarctic subtidal marine benthic communities [11–12], despite the limitations constraining data collection on the continent (e.g., limitations of scuba diving time, year-round accessibility, and duration of research projects). Nevertheless, long-term data is still needed to elucidate the dynamics of Antarctic marine benthic organism [7]. Long-term observations of marine benthic communities are essential for a detailed understanding and predictions of the dynamics of the marine benthic ecosystem [11].

The species composition and assemblage structure of marine benthic organisms are strongly related to environmental conditions and can therefore be used as an effective tool to identify the impacts of various environmental factors [13–15]. Macroalgal community structure has also been highlighted as a good indicator of environmental changes caused by natural or anthropogenic processes in marine coastal waters [16–18]. In particular, macroalgal tissue nitrogen content can be used as a potential indicator to evaluate nutrient enrichment in the water column [19]. Additionally, marine benthic organisms play a significant role in the flux of organic matter in the Antarctic sublittoral ecosystem [20]. Therefore, a systematic study of marine benthic community structure will help to better understand and predict the patterns of distribution and organization of benthic communities and their vulnerability to change.

Antarctic marine ecosystems are highly responsive to natural and human-driven perturbations [21]. Strom-induced wave action and ice effects are considered major disturbances in Antarctic shallow subtidal zones [21]. The impacts of human activities related to the construction, operation, and maintenance of the research station on Antarctic environment, potentially influencing the marine ecosystem, have been reported from the past decade [9,22]. The Jang Bogo Antarctic Research Station is the second permanent year-round research base established by the Republic of Korea. The station is located in Victoria Land on the eastern flank of the Ross Sea rift, part of the West Antarctic rift system, which is one of several large tectonic provinces of the Earth formed by the Cretaceous to Cenozoic extension [23]. The Jang Bogo Station (including the dock) was constructed over 2 years (2012–2014) and has been operational since February 2014. During the construction of dock, an amount of gravels and boulders, by-product of grading and excavation for the mat foundation of the Jang Bogo Station was used for backfilling in stainless steel boxes (2012–2013, S1 Fig). Precast concrete blocks were also used to the top of boxes. Although the excavation site was contained within a turbidity barrier to avoid dispersion of suspended materials, an unknown amount of sediment was released into the nearby coastal area during construction of the station and dock. In addition, during operations, a small quantity of wastewater and sewage is discharged into the near-shore area in front of the research station. Thus, this study allowed a unique opportunity to examine the responses of marine benthic community to the disturbance and its recovery pattern.

In the present study, we describe marine benthic communities collected from the study area near the Jang Bogo Antarctic Research Station in Terra Nova Bay, Antarctica, during the 2012–2018 austral summer seasons. The main objective of the study was to characterize the composition, structure and variability of the marine benthic community to better understand and predict its dynamics. This study will also contribute to the establishment of a long-term monitoring program of the subtidal community and enhance the ability to detect changes in marine benthic organisms caused by natural and human-driven activities. This study represents a comprehensive examination of long-term patterns of the subtidal benthic community at the study area near the Jang Bogo Antarctic Research Station, Terra Nova Bay, and results will improve our knowledge of the marine benthic communities of Antarctica.

## Materials and methods

### Study area

**Fig 1:**
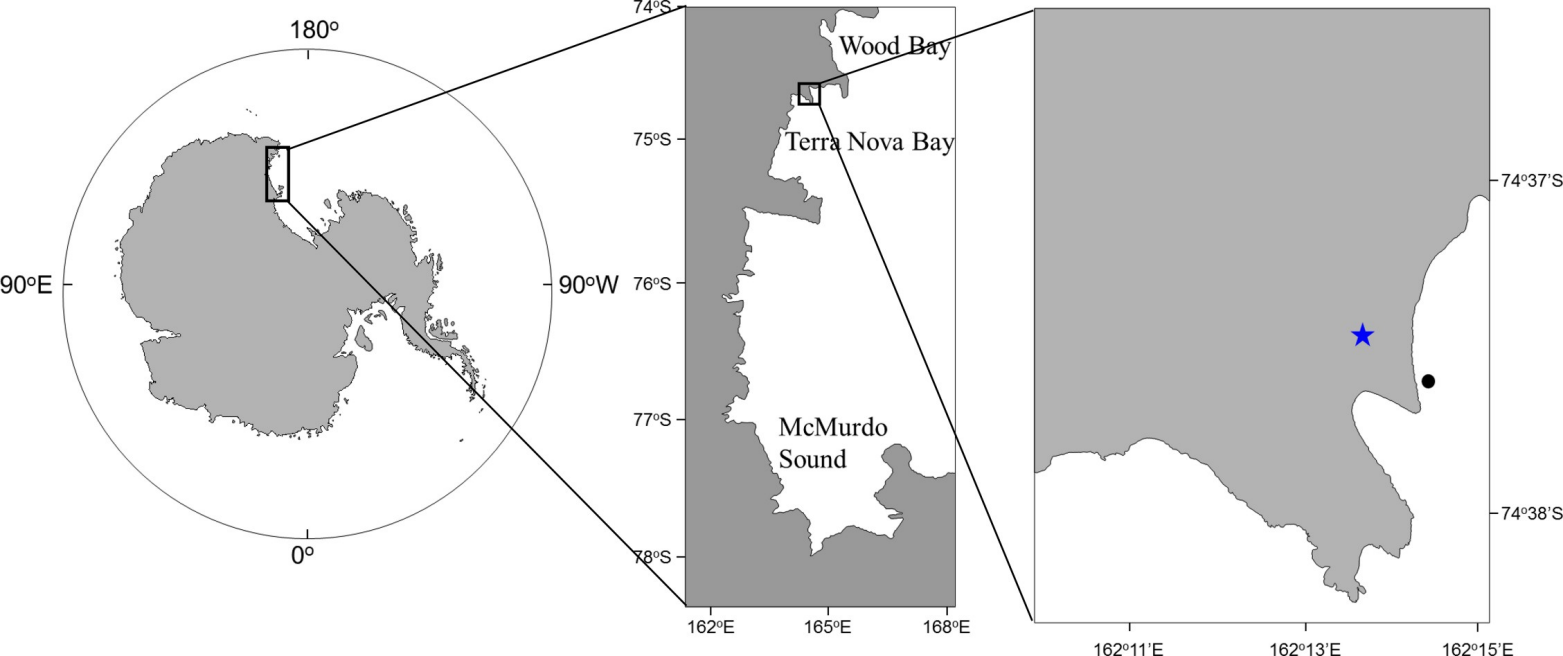
The study area and sampling site near the Jang Bogo Antarctic Research Station in Terra Nova Bay, Antarctica. Star indicates the Jang Bogo Station.

The study site was located in Terra Nova Bay, a coastal zone at the southernmost edge of North Victoria Land between Cape Washington and the Drygalski Ice Tongue (74°37’S, 164°14’W) (Fig 1). The study site was located approximately 0.6 km from the Jang Bogo Antarctic Research Station and was therefore potentially affected by the construction of the station. This site has a rocky shore composed of a mixture of hard bedrock and compacted cobbles, with some isolated patches of silt. Diatoms (*Fragilariopsis* spp.) and amphipods dominate the region from the intertidal zone to the shallow subtidal zone. Red algae [e.g., *Iridaea cordata* (Turner) Bory de Saint-Vincent, 1826 and crustose coralline algae], soft coral, Antarctic scallop [*Adamussium colbecki* (Smith, 1902)] and sponges were observed at 5–20 m water depth in this area. The mean annual air temperature was ‒14.6°C from 2010–2013 [24]. Water temperature and salinity were relatively constant throughout the experimental period, with means of ‒1.70°C and 33.48, respectively. Field researches in this area were permitted by the Ministry of Foreign Affairs, Republic of Korea.

### Experimental design and sampling

The survey was performed over the 2012–2018 summer seasons (January–February) by SCUBA and digital imaging. To characterize annual changes in marine benthic assemblage composition, three 30-m-long transect lines were permanently established at preselected angles (45, 90 and 135 degrees) to the shoreline at the study site. The distance between transects at the starting point at the nearshore end was approximately 30 m. The range of water depth covered by the transects varied with the topography of the site; the transects began at a depth of 5–6 m and ended at depths between 12 and 16 m. Additional detailed information for transect lines is shown in S1 Table. A Nikon D800 digital camera equipped with a wide-angle lens and Nauticam underwater housing was used to obtain the images. First, we conducted video surveys along the transects and took a picture of each quadrat using the digital camera. Then, the camera was positioned 1.5 m above a permanent 1 × 1 m (1 × 0.5 m in 2014) quadrat every 6 m along the transect, providing six quadrats per transect. Eighteen 1 × 1 m quadrats (twelve 1 × 0.5 m quadrats in 2014) were taken for the study area. For percent cover of benthic organisms, a quadrat with 400 subplots was overlaid on an image using Adobe Photoshop CS6 software (Adobe Systems Inc., San Jose, CA, USA). Some species were collected for species identification. Species identification was determined using the following literature: Blake [25], Brueggeman [26–28], Burton [29], Campos et al. [30], Cano and López-González [31], Cantone [32], Cefarelli et al. [33], Choe et al. [34], Clark [35], Clarke and Johnston [36], Cormaci et al [37], Desqueyroux-Faúndez [38], Galea and Schories [39], Ghiglione et al. [40], Gibson [41], Göcke and Janussen [42], Hasle [43], Hayward [44], Schories and Kohlberg [45], Koltun [46], Larson [47], McKnight [48], Ríos et al. [49], Topsent [50], Verseveldt and Ofwegen [51], Vine [52], Niell [53]. The percent cover of each species was estimated for each subplot using a visual estimation method, which has been shown to be more efficient and accurate than the random-point quadrat method [54]. The relative coverage (RC) of each species was determined as follows: RC = [percentage cover of one species/percentage cover of all species in a quadrat] × 100.

### Data analyses

All data are presented as mean ± standard error. To compare the structure and diversity of marine benthic communities among sampling times, Margalef’s Richness index (*R*), Pielou’s Evenness index (*J*′), Shannon’s Diversity index (*H*′), Simpson’s Dominance index (*D*) and *K*-dominance curves were calculated using the PRIMER 6.0 software package (PRIMER-E Ltd., Plymouth, UK). Similarity in species composition was analyzed using the Bray-Curtis similarity coefficient. Cluster analysis was conducted using a hierarchical method with group-average linking, and non-metric multidimensional scaling (nMDS) was performed to compare marine benthic assemblages among sampling times. Cluster analysis and nMDS were analyzed using PRIMER 6.0 software.

Significant differences in diversity indices (*R*, *J*′, *H*′ and *D*) were analyzed using two-way ANOVA with one fixed factor (year) and one random factor (transect line). Data were tested for normality and homogeneity of variance to meet the assumptions of parametric statistics prior to ANOVA analysis. Because these assumptions were not satisfied, data were log-transformed. When significant differences among treatments were observed, a Student-Newman-Keuls (SNK) post-hoc test was performed. Statistical significance was set at alpha < 0.05. All ANOVA analyses were performed using IBM SPSS Statistics 20.0 (IBM Corporation, Armonk, NY, USA).

## Results

### Marine benthic community structure

**Fig 2:**
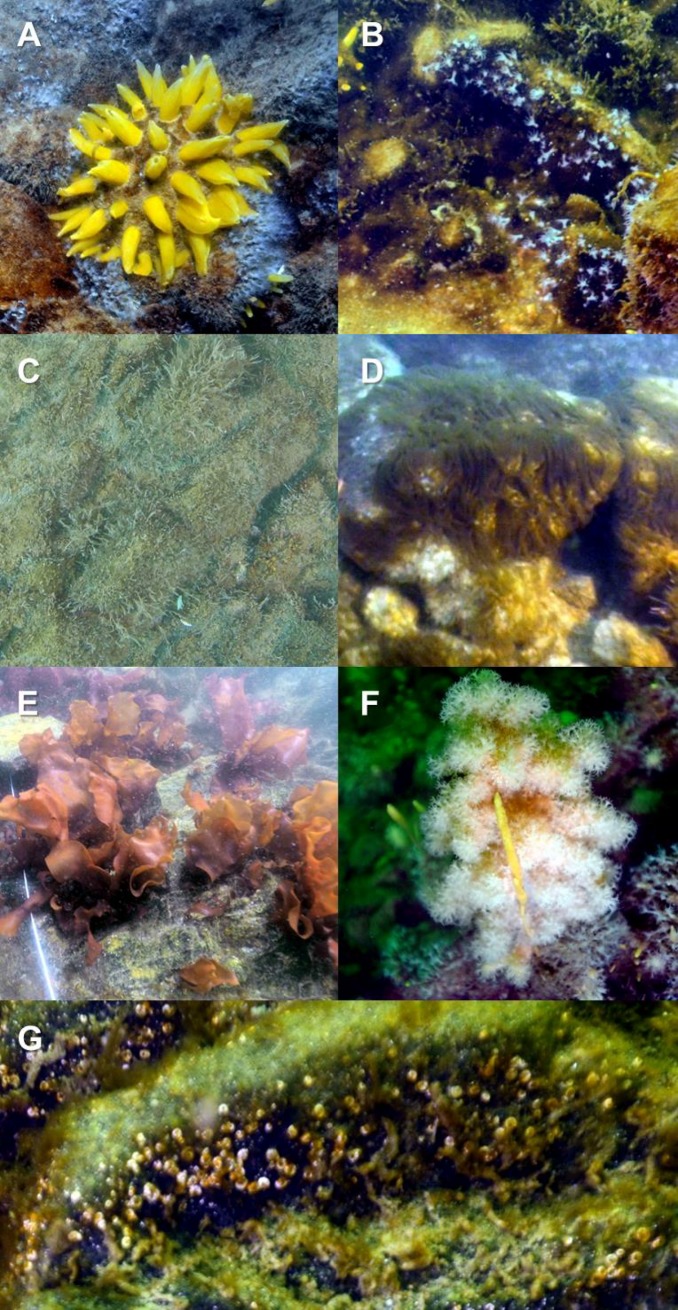
Marine benthic organisms at the study site in Terra Nova Bay. (A) *Sphaerotylus antarcticus* Kirkpatrick, 1907, (B) *Clavularia frankliniana* Roule, 1902, (C) *Hydractinia* sp., (D) *Fragilariopsis* spp., (E) *Iridaea cordata* (Turner) Bory de Saint-Vincent, 1826, (F) *Alcyonium antarcticum* Wright& Studer, 1889, and (G) *Metalaeospira pixelli* (Harris, 1969). Photographs were taken in January–February 2012–2018 at 6–16 m water depths.

The video survey indicated that a total of 26 taxa were identified in the study area during the experimental period (Table 1). The community was composed of 3 taxa of algae and 23 taxa of invertebrates. Fig 2 presents the dominant species [*Sphaerotylus antarcticus* Kirkpatrick, 1907, *Clavularia frankliniana* Roule, 1902, *Hydractinia* sp., *Fragilariopsis* spp., *Iridaea cordata*, *Alcyonium antarcticum* Wright& Studer, 1889, and *Metalaeospira pixelli* (Harris, 1969)] at the study area.

**Table 1:** List of species observed at the study area in Terra Nova Bay during the experimental period, 2012–2018, via video survey.

Taxa	
**Porifera**	**Cnidaria**
*Dendrilla antarctica* Topsent, 1905	*Urticinopsis antarctica* (Verrill, 1922)
*Isodictya setifera* (Topsent, 1901)	*Alcyonium antarcticum* Wright & Studer, 1889
*Homaxinella balfourensis* (Ridley & Dendy, 1886)	*Clavularia frankliniana* Roule, 1902
*Haliclona tenella* (Lendenfeld, 1887)	*Hydractinia* sp.
*Polymastia invaginata* Kirkpatrick, 1907	*Diplulmaris antarctica* Maas, 1908
*Sphaerotylus antarcticus* Kirkpatrick, 1907	**Mollusca**
*Inflatella belli* (Kirkpatrick, 1907)	*Laternula elliptica* (P. P. King, 1832)
**Nemertea**	*Neobuccinum eatoni* (E. A. Smith, 1875)
*Parborlasia corrugatus* (McIntosh, 1876)	*Tritoniella belli* Eliot, 1907
**Echinodermata**	**Annelida**
*Odontaster validus* Koehler, 1906	*Metalaeospira pixelli* (Harris, 1969)
*Diplasterias brucei* (Koehler, 1907)	**Ochrophyta**
*Acodontaster hodgsoni* (Bell, 1908)	*Fragilariopsis* spp.
*Ophiosparte gigas* Koehler, 1922	**Rhodophyta**
*Sterechinus neumayeri* (Meissner, 1900)	Crustose coralline algae
**Arthropoda**	*Iridaea cordata* (Turner) Bory de Saint-Vincent, 1826
*Ammothea clausi* Pfeffer, 1889	

In the quadrats of the three transect lines, the dominant organisms varied throughout the year, and their total coverage increased over the period from 2012–2018, with the exception of 2014 (Table 2). In 2012, during the construction of the Jang Bogo Station, total coverage of marine organisms was very low (average 28.1 ± 1.9%), while sediment covered 57.4 ± 7.4%. *Sphaerotylus antarcticus* was the dominant species, with relative coverage of more than 76.9 ± 13.0%. Only three other species [*Polymastia invaginata*, *Laternula elliptica* (P. P. King, 1832), and *Odontaster validus* Koehler, 1906] were observed at more than 1% coverage (4% relative coverage). In 2014, after completion of the Jang Bogo Station, the diatom *Fragilariopsis* spp. rapidly increased and became the dominant species in this area. The coverage and relative coverage of diatoms were 62.4 ± 1.1 and 87.4 ± 2.8%, respectively. Thus, the marine benthic assemblage was very simple during the 2014 summer season. During the period of 2015–2018, total coverage of marine organisms gradually increased, ranging from 50.4 ± 2.7 to 86.5 ± 2.0%. Although the coverage of diatom mats dramatically decreased compared to 2014, *Fragilariopsis* spp. was still the most abundant organism. Additionally, the number of species increased over the period of 2015–2018. *Sphaerotylus antarcticus*, *Clavularia frankliniana*, and *Hydractinia* sp. exhibited increases in percent cover during this time period.

**Table 2:** List of species with coverage (C) and relative coverage (RC) in Terra Nova Bay during 2012–2018.

Species	2012		2014		2015		2016		2017		2018	
	C	RC	C	RC	C	RC	C	RC	C	RC	C	RC
**Porifera**												
*Dendrilla antarctica*											+	+
*Homaxinella balfourensis*							+	+	+	+	1.0	1.2
*Polymastia invaginata*	1.6	5.7							1.0	1.3	3.6	4.2
*Sphaerotylus antarcticus*	21.6	76.9	3.8	5.3	3.3	7.6	3.6	6.5	2.3	2.8	18.8	21.8
*Inflatella belli*									+	+		
**Cnidaria**												
*Alcyonium antarcticum*	+	1.1			+	1.6	+	+	+	+	+	+
*Clavularia frankliniana*			+	+	3.6	7.2	4.1	7.4	+	+	22.7	26.3
*Hydractinia* sp.					19.7	39.2	24.2	45.3	24.3	30.0		
**Mollusca**												
*Laternula elliptica*	2.2	7.8			+	+			1.1	1.4	+	+
*Neobuccinum eatoni*									+	+		
*Tritoniella belli*									+	+		
**Nemertea**												
*Parborlasia corrugatus*									+	+		
**Annelida**												
*Metalaeospira pixelli*			1.6	2.3	+	+	2.9	6.0	2.2	2.7		
**Echinodermata**												
*Odontaster validus*	1.2	4.3	+	+	+	+					+	+
*Diplasterias brucei*	+	1.8			+	+					+	+
*Acodontaster hodgsoni*									+	+		
*Ophiosparte gigas*	+	+			+	+	+	+	+	1.1	+	+
*Sterechinus neumayeri*									+	+	+	+
**Ochrophyta**												
*Fragilariopsis* spp.			62.4	87.4	20.9	41.4	14.6	28.0	47.6	58.6	38.4	44.4
**Rhodophyta**												
*Iridaea cordata*	+	2.1	2.6	3.2	1.6	3.1	3.3	6.1	+	+		
**Total coverage (%)**	28.1		71.6		50.4		53.2		81.1		86.5	
**Total number of species**	8		6		11		9		17		12	
**Sediment**	57.4		0.0		0.0		+		0.0		11.8	

+ indicates that coverage was less than 1%

### Dominance curves and diversity indices

**Fig 3:**
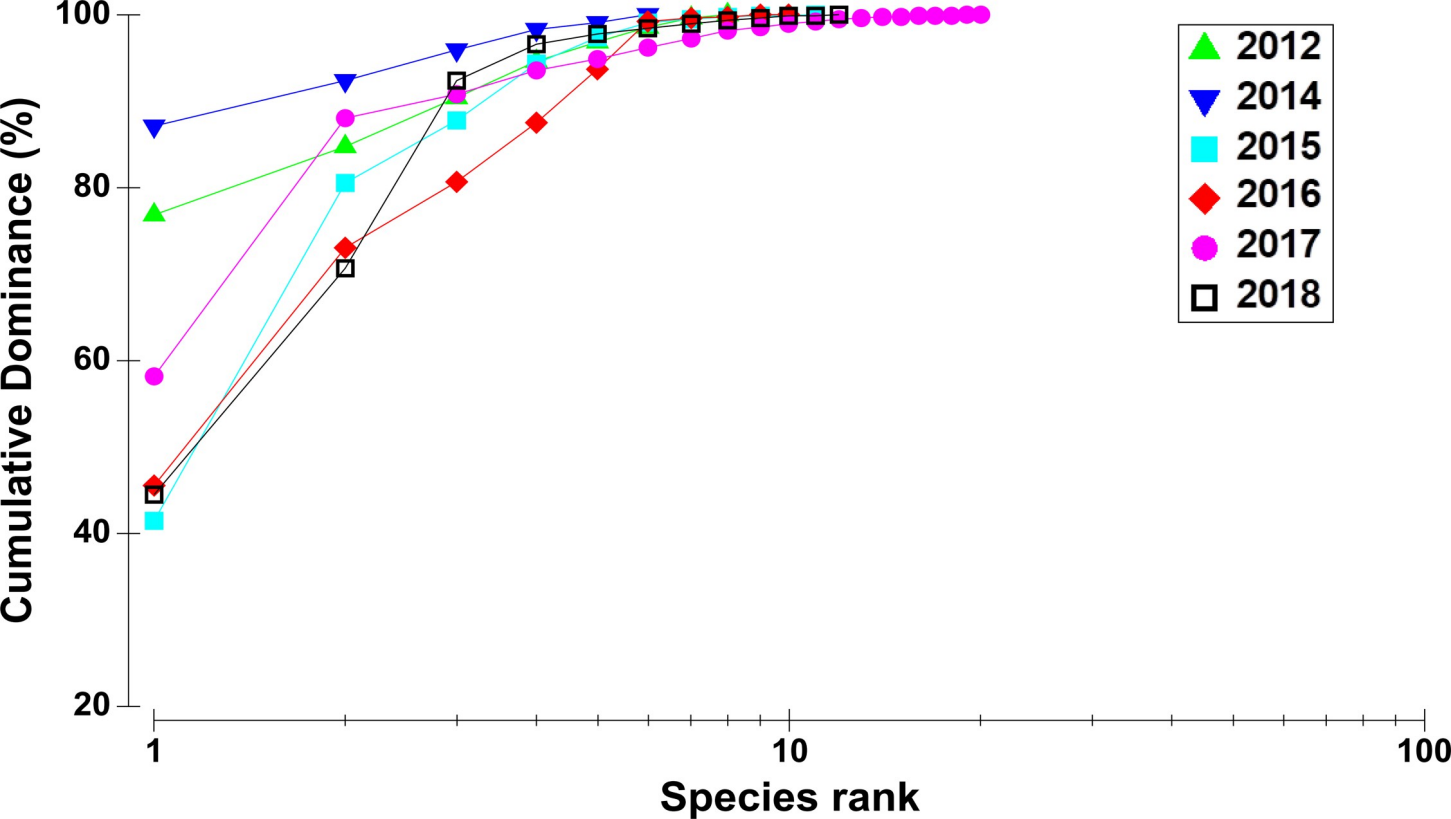
*K*-dominance curves for coverage of marine benthic organisms in each year at the study site in Terra Nova Bay, Antarctica.

**Fig 4:**
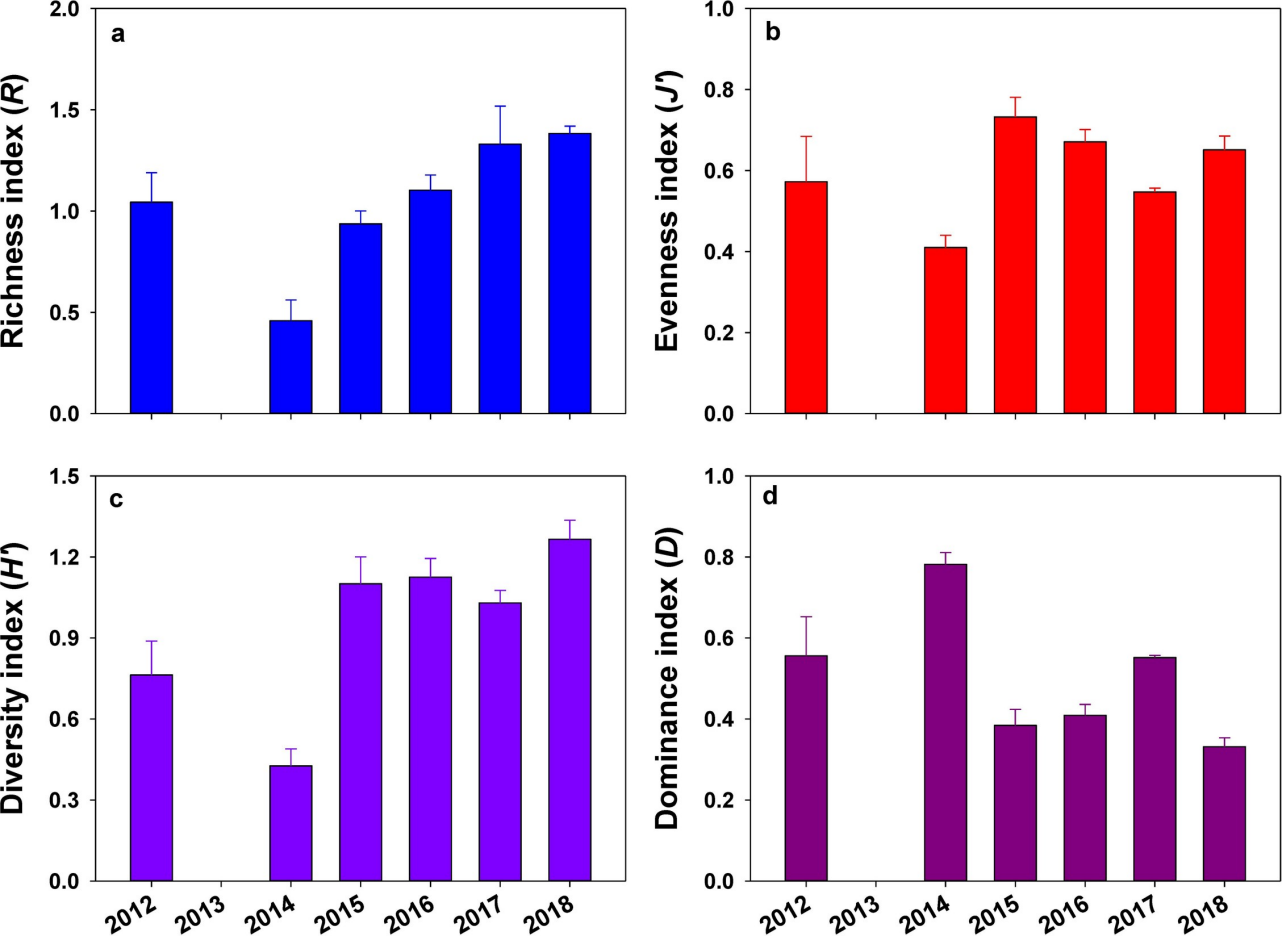
Diversity indices. Margalef’s Richness index (*R*; a), Pielou’s Evenness index (*J’*; b), Shannon’s Diversity index (*H’*; c) and Simpson’s Dominance index (*D*; d) of marine benthic organisms at the study site in Terra Nova Bay, Antarctica. Values are means ± SE (n = 12–18). The arrow indicates the construction period of Jang Bogo station (time of disturbance).

The shape of cumulative species dominance plots (*K*-dominance curves) differed among sampling times (Fig 3). In 2012 and 2014, the community was dominated by a single species or group (*Sphaerotylus antarcticus* or *Fragilariopsis* spp.), and the *K*-dominance curves were gently sloped. In contrast, the 2015–2018 curves showed dominance by several species with diagonal slopes. The species richness index, evenness index, diversity index, and dominance index varied significantly (*P* < 0.001 in all cases) with sampling time (Fig 4). However, difference in diversity indices among transect lines and sampling time × transect line interactions were not significant (Fig 4). Species richness, evenness, and diversity were lowest in 2014, when the dominance index was the highest due to the dominance of the diatom mat. Species richness, diversity, and evenness gradually increased in subsequent years, often reaching peak values, compared to values in 2014. However, the dominance index was lowest during the 2015–2018 period.

### Cluster analysis and nMDS

**Fig 5:**
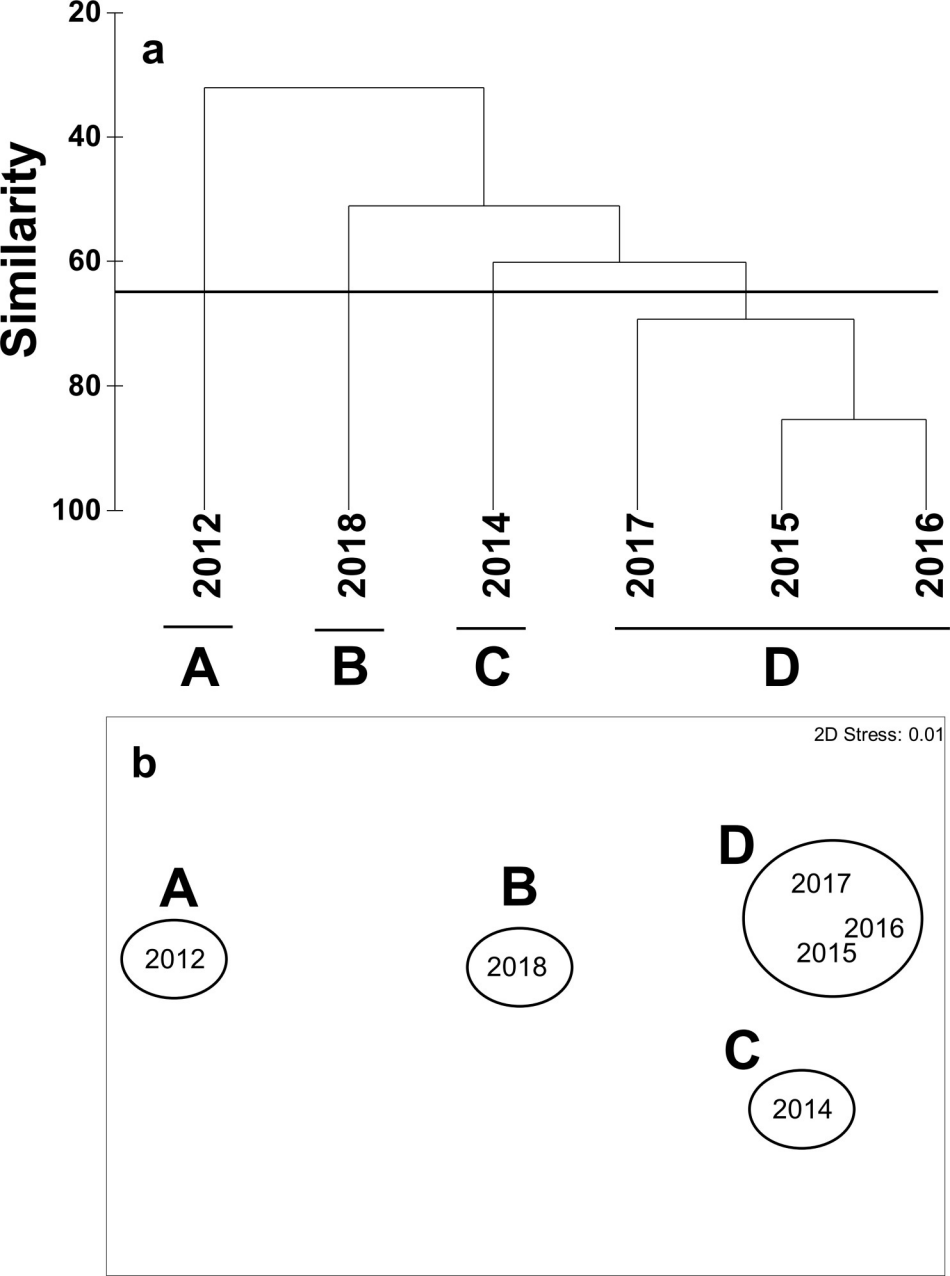
Cluster analysis and nMDS ordination of marine organism coverage. Dendrogram of the hierarchical clustering of marine benthic assemblages (similarity threshold, 65%) based on Bray-Curtis similarities (a) and nMDS plot (b).

The cluster analysis and nMDS ordination of marine organism coverage produced four clusters (A–D, with a stress value of 0.01) at a similarity of 65% (Fig 5). Clusters A and C exhibited high relative coverage and were composed of sponges or diatoms. Cluster B had high abundances of sponges, soft coral, and diatoms, with relatively high abundance and diversity. Cluster D consisted of quadrats measured in 2015–2017. This group was characterized by high abundances of sponge, soft coral, hydroids and diatoms, with high values of diversity.

## Discussion

### Composition and structure of the benthic community

Since the 1990s, many attempts have been made to characterize Antarctic marine benthic community structure, primarily at the Antarctic Peninsula [55–58]. In addition to this region, several studies of the composition and community structure of marine benthic organisms have been conducted over several years in Terra Nova Bay, between Campbell Glacier Tongue and Drygalski Ice Tongue, on Victoria Land of southeast Antarctica [11,59–61]. However, most studies have focused on relatively deep habitats [62,63]. Therefore, minimal information exists regarding the distribution and community structure of Antarctic marine benthic organisms in coastal areas that are directly affected by natural or anthropogenic impacts. To our knowledge, this study is the first to report long-term observations of the composition and community structure of marine benthic organisms and natural recovery processes in Terra Nova Bay, with a specific focus on areas where human impacts may have occurred.

The overall community structure and assemblage of marine benthic organisms in this area did not differ greatly from those previously reported in a coastal area adjacent to the Italian research station in Terra Nova Bay [59,60]. In the present study, the diatom taxa (*Fragilariopsis* spp.) were the most abundant species in the study area over the experimental period, even though they temporarily disappeared in 2012 due to the construction of the Jang Bogo station. These diatoms were found attached to benthic organisms, rocks, or the soft bottom. The planktonic and ice-associated diatoms such as *Fragilariopsis curta* and *F*. *nana* were found in Terra Nova Bay in January [64]. A few studies reported that summer diatom-dominated bloom in Terra Nova Bay were related with the ice edge recession [65,66]. Both *F*. *curta* and *F*. *nana* were observed in the benthic diatom community [67]. Diatoms can function as the most important primary producers in Polar Regions during certain periods of the year [68–70]. Their role as primary producers is particularly strong in Antarctic shallow subtidal zones that are devoid of macrophytes. For example, benthic diatoms accounted for around 40% of total benthic primary production (60% derived from seaweeds) in Young Sound, a high Arctic fjord, indicating an ecologically crucial role in trophic relationships [71]. Benthic diatoms also play an essential role in the biogeochemical cycles of carbon, nitrogen, phosphorus, and silica [72]. In this study, the coverage and relative coverage of diatoms, *Fragilariopsis* spp., were both nearly 50% during the austral summer period of 2017–2018. Furthermore, this diatom species appeared to account for a significant amount of biomass (Fig 1D), indicating that it serves a vital role in maintaining the marine benthic community and controlling biogeochemical cycling.

In contrast, *Iridaea cordata* was the only macroalgal species inside quadrats at the study area and occurred at very low abundance compared to previous findings [7]. Furthermore, several species (e.g., *Polymastia invaginata*, *Sphaerotylus antarcticus*, *Clavularia frankliniana*, *Hydractinia* sp., and *Metalaeospira pixelli*) previously found in Terra Nova Bay or deep water were present at the study area [40,63,73–75]. Additionally, our video survey revealed the presence of crustose coralline algae near the study area; however, its coverage and abundance were negligible. Species number was relatively low (average of 10 species) compared to previous reports from Terra Nova Bay and the Antarctic Peninsula [37,60,76,77]. The evenness index ranged from 0.41 to 0.73 throughout the study period, with an average of 0.60. This value was similar to that observed by [78], who reported that the evenness index of marine benthic organisms in Terra Nova Bay ranged from 0.03 to 0.72. In the present study, the average diversity index was 0.95, which was lower than that observed at other study areas in Terra Nova Bay [78]. Considering the challenges associated with the quantitative research process in the Antarctic region, these diversity indices will help to better understand the community structure of marine benthic organisms in this area.

### Responses of benthic community structure to disturbances

Antarctic marine benthic communities are among the most stable ecosystems in the world, and the distribution, composition, and characteristics of marine species reflect their adaptation to polar environments [60,79–81]. However, these communities are often exposed to various disturbances such as extreme wave action, temperature increases, ocean acidification, and inflow of wastewater and sewage, and marine benthic communities are among the most susceptible due to their adaptations to the severe limitations associated with harsh environmental conditions [8, 82]. Additionally, Antarctic marine benthic ecosystems have slow recovery rates from disturbance due to the slow pace of reproduction, colonization, and growth [21]. The pre-disturbance data of benthic community structure or undisturbed site (reference or control site) is required to evaluate the post-disturbance recovery of benthic community structure. Actually, benthic structure community was monitored from 2011 to compare benthic community structure before and after the disturbance. However, we did not find previous study sites in 2012 due to the difficulty of accessibility. The data of benthic community structure collected in 2011 could be used to compare the marine benthic community structure before and after the disturbance. Unfortunately, it may be difficult to precisely assess the recovery rate of marine benthic community structure. Nevertheless, this study provided valuable information towards understanding the sequential recovery process of Antarctic marine benthic community structure after the disturbance. In the present study, recovery of the marine benthic community gradually occurred over the 5-year period following disturbance. Species number and diversity indices were nearly constant during 2015–2018. However, the results of the nMDS ordination of species coverage revealed that community structure in 2015–2017 differed from that in 2018, indicating that community structure is still recovering in this area. Thus, further research is required to elucidate the post-disturbance settlement mechanisms of marine benthic organisms at the study area in Terra Nova Bay.

In the present study, initial increases in sediment coverage and declines of diatoms were caused by the introduction of sediment into the coastal regions due to the construction of the Jang Bogo Station including the dock. Only *Sphaerotylus antarcticus*, a common Antarctic sponge, exhibited the highest values of coverage and relative coverage. There was a large cyclone spinning in the Ross Sea in January 2014. In February 2014, one month after the disturbance, the sediment was not observed and total coverage of all marine organisms except diatoms seriously declined (< 10%) at the study area. This suggested that strong wave action induced by a large cyclone had a negative impacts on marine benthic community (S1 Fig). Storm-induced wave can have a strong impacts on benthic communities above approximately 12 m [21]. It leads to mechanical abrasion of marine organisms by either directly or by moving boulders around [83]. Additionally, strong wave results in the change of the distribution of meiofauna and small macrofauna [84].

However, the coverage of diatoms dramatically increased in 2014 and it resulted the increase of *Hydractinia* sp. during 2015–2017. *Hydractinia* eats masses of diatoms as a food resource and is able to consume up to several times its own size [74]. From 2015 to 2018, total coverage, species number, the richness index, evenness index, and diversity index steadily increased, while the dominance index was low. This would indicate that diatoms may accelerate and facilitate the rate of recruitment and settlement of other organisms. According to three models of succession proposed by [85], the settlement or colonization of diatoms and bacteria, which are collectively referred to as biofilm, in an open space created by disturbance is the initial step of succession. Subsequently, this biofilm establishment facilitates the settlement of invertebrates and macroalgae [86]. Additionally, the settlement of marine sessile animals can be enhanced by the presence of other animals [87]. Thus, diatoms play crucial roles in the recovery pattern of marine benthic organisms in Antarctica as well as in temperate or tropical regions.

In conclusion, image analysis from underwater video transects in Terra Nova Bay demonstrated that the community structure and assemblage of marine benthic organisms were relatively simple, and that diversity indices were similar or lower compared to data previously reported from Terra Nova Bay. The diatom *Fragilariopsis* spp., the sea ice-related species, was the dominant taxa, indicating its important role in the recovery process of the marine ecosystem of this area following disturbance. Although the coverage of diatoms quickly recovered, leading to an accelerated rate of recruitment and facilitation of the settlement of other organisms following disturbance, the marine benthic community slowly recovered over a long period of time. Considering that Antarctic marine benthic ecosystems exhibit slow recovery rates from disturbance, long-term monitoring of the marine ecology of benthic communities is required in Terra Nova Bay.

## Supporting information

S1 Fig The construction of dock near the Jang Bogo Antarctic Research Station during 2012–2013 (A and B) and the dock disturbed by storm-induced strong wave action in January 2014 (C and D).
(TIF)

S1 Table Transect line position (latitude, longitude and depth) at the study area near the Jang Bogo Antarctic Research Station in Terra Nova Bay, Antarctica. (DOC)
