# Supplementary figures and images for: Composition and structure of the marine benthic community in Terra Nova Bay, Antarctica: Responses of the benthic assemblage to disturbances
Source: PLoS One. 2019 Dec 2;14(12):e0225551. doi: 10.1371/journal.pone.0225551 (PMC6886853; doi:10.1371/journal.pone.0225551)

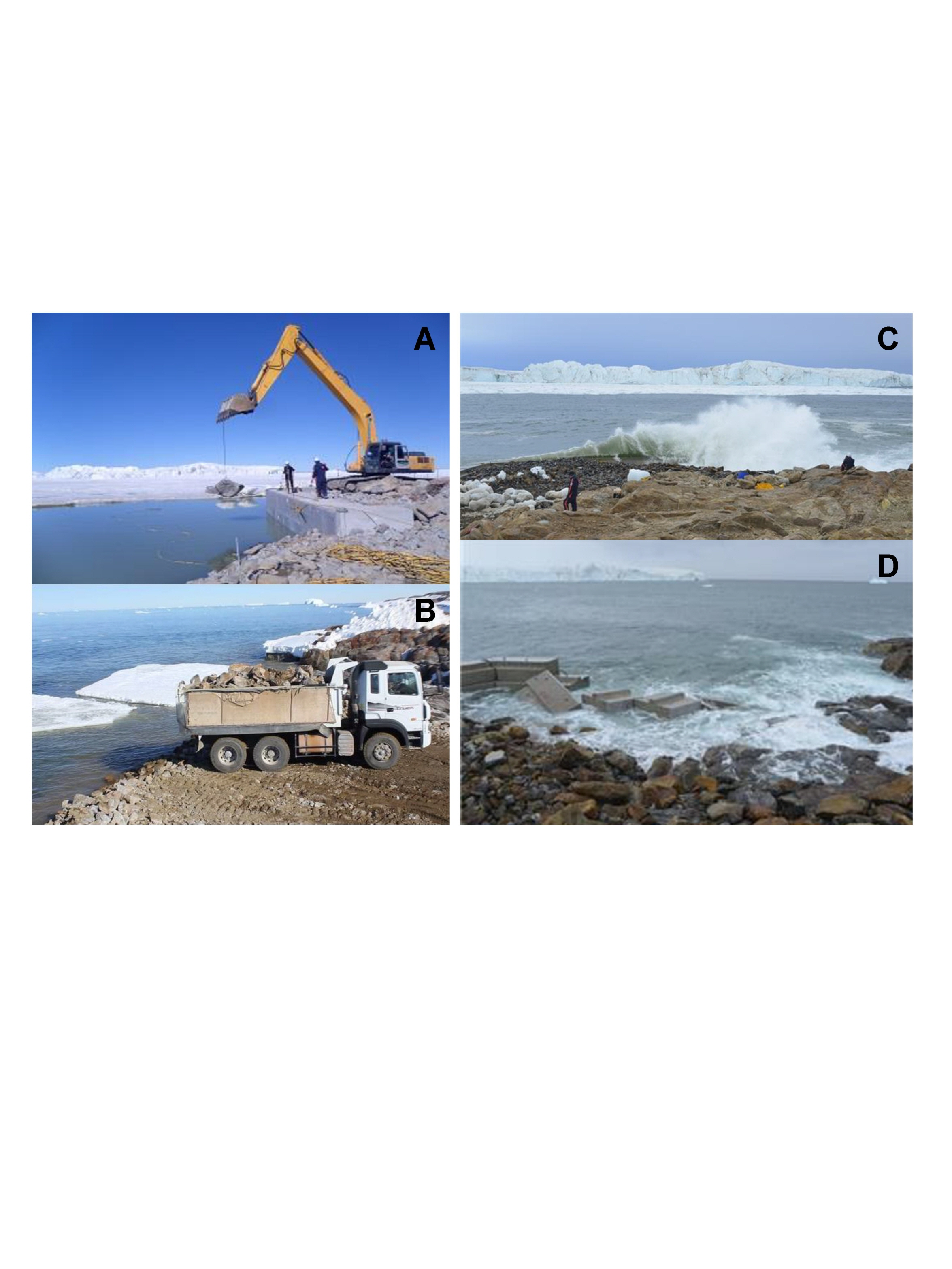

Supplement: S1 Fig — The construction of dock near the Jang Bogo Antarctic Research Station during 2012–2013 (A and B) and the dock disturbed by storm-induced strong wave action in January 2014 (C and D). (TIF) [file pone.0225551.s001.tif]
