# Supplementary material for: Composition and structure of the marine benthic community in Terra Nova Bay, Antarctica: Responses of the benthic assemblage to disturbances
Source: PLoS One. 2019 Dec 2;14(12):e0225551. doi: 10.1371/journal.pone.0225551 (PMC6886853; doi:10.1371/journal.pone.0225551)
Supplement: S1 Table — (DOC) [file pone.0225551.s002.doc]

S1 Table.

| **Transect label** | **Starting point (GPS)** | **End point (GPS)** | **Depth (m)** |
| --- | --- | --- | --- |
| KOPRI_JBG_TA | 74°37’38.5”S  164°14’26.2”E | 74°37’37.1”S  164°14’27.5E | 5–12 |
| KOPRI_JBG_TB | 74°37’39.1”S  164°14’26.9”E | 74°37’38.9”S  164°14’33.5”E | 6–16 |
| KOPRI_JBG_TC | 74°37’39.8”S  164°14’27.0”E | 74°37’40.6”S  164°14’33.7”E | 6–16 |
